# Protein adducts with lipid peroxidation products in patients with psoriasis

**DOI:** 10.1016/j.redox.2023.102729

**Published:** 2023-05-03

**Authors:** Adam Wroński, Agnieszka Gęgotek, Elżbieta Skrzydlewska

**Affiliations:** a Dermatological Specialized Center "DERMAL" NZOZ in Bialystok, Poland; b Department of Analytical Chemistry, Medical University of Bialystok, Poland

**Keywords:** Psoriasis, Proteins, Lipid peroxidation, 4-Hydroxynonenal, Malondialdehyde, Adducts

## Abstract

Psoriasis, one of the most frequent immune-mediated skin diseases, is manifested by numerous psoriatic lessons on the skin caused by excessive proliferation and keratinization of epidermal cells. These disorders of keratinocyte metabolism are caused by a pathological interaction with the cells of the immune system, including lymphocytes, which in psoriasis are also responsible for systemic inflammation. This is accompanied by oxidative stress, which promotes the formation of lipid peroxidation products, including reactive aldehydes and isoprostanes, which are additional pro-inflammatory signaling molecules. Therefore, the presented review is focused on highlighting changes that occur during psoriasis development at the level of lipid peroxidation products, including 4-hydroxynonenal, 4-oxononenal, malondialdehyde, and acrolein, and their influence on protein structures. Furthermore, we will examine inducing agents of cellular functioning, as well as intercellular signaling. These lipid peroxidation products can form adducts with a variety of proteins with different functions in the body, including proteins within skin cells and cells of the immune system. This is especially true in autoimmune diseases such as psoriasis. For example, these changes concern proteins involved in maintaining redox homeostasis or pro-inflammatory signaling. Therefore, the formation of such adducts should attract attention, especially during the design of preventive cosmetics or anti-psoriasis therapies.

## Highlights

• Lipid peroxidation product-protein adducts results from redox imbalance.
• Adducts may act as signaling molecules in psoriasis development.
• Adducts induce also pro-inflammatory processes in the skin and blood of patients.
• Counteracting 4-HNE-protein adducts formation may be important in therapy of psoriasis.

## Graphical abstract

**Figure:**
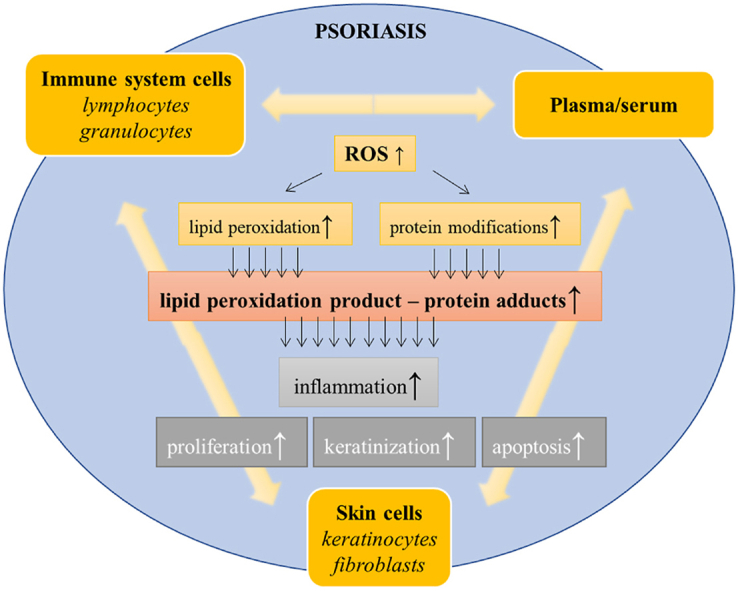

## Abbreviations:

4-HNE: 4-hydroxynonenal
4-ONE: 4-oxononenal
AOPP: advanced oxidation protein product
AP-1: activator protein 1
Bach1: BTB and CNC homology 1
CAT: catalase
CBD: cannabidiol
COX: cyclooxygenase
DC: dendritic cell
GRO: growth-regulated oncogene
GSH-Px: glutathione peroxidase
GSSG-R: glutathione reductase
IFN: interferon
IL: interleukin
IκB: inhibitor of NFκB
iNOS: inducible nitric oxide synthase
Keap1: kelch-like ECH-associated protein 1
KGF: keratinocyte growth factor
LDL: low-density lipoprotein
LHP: lipid hydroperoxide
LOX: lipoxygenase
MAPK: mitogen-activated protein kinase
MDA: malondialdehyde
MG-protein: methylglyoxal-proteins
NFκB: nuclear factor κB
NQO1: NAD(P)H quinone oxidoreductase 1
Nrf2: nuclear factor erythroid 2–related factor 2
p21: protein 21
p62: protein 62
PASI: psoriasis area severity index
PCC: protein carbonyl compounds
PDGF: platelet-derived growth factor
PKC: protein kinase C
PLA2: phospholipase A2
PP: pyrrolized protein
PPARδ: peroxisome proliferator-activated receptor δ
PUFA: polyunsaturated fatty acid
ROS: reactive oxygen species
SOD: superoxidase dismutase
TAS: total antioxidant status
Th17: T helper 17 cells
TNFα: tumor necrosis factor α
Trx-R: thioredoxin reductase
VEGF: vascular endothelial growth factor
VMAT2: vesicular monoamine transporter 2

## 1 Introduction

**Fig. 1:**
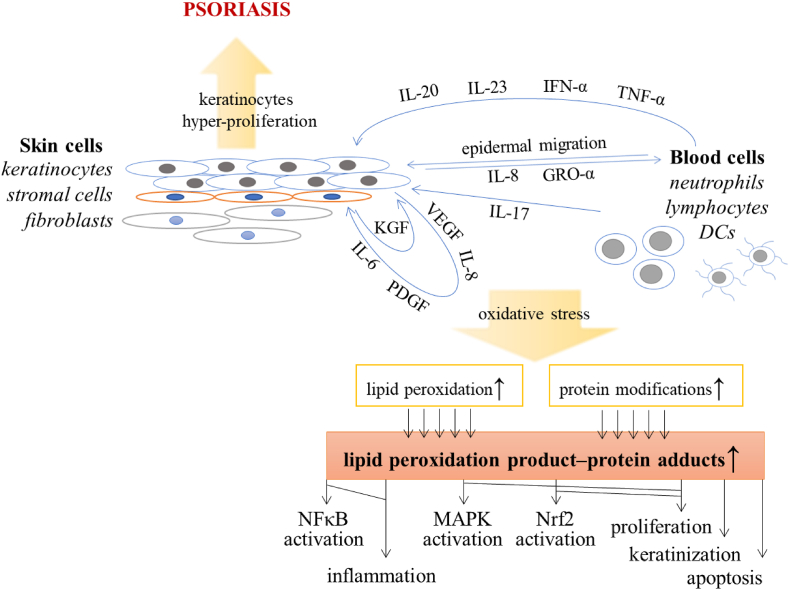
Scheme of changes and interactions occurring during the psoriasis development. Abbreviations: DC, dendritic cell; GRO, growth-regulated oncogene; IFN, interferon; IL, interleukin; KGF, keratinocyte growth factor; MAPK, mitogen-activated protein kinase; NFκB, nuclear factor κB; Nrf2, nuclear factor erythroid 2–related factor 2; PDGF, platelet-derived growth factor; TNFα, tumor necrosis factor α; VEGF, vascular endothelial growth factor.

Psoriasis is one of the most frequent immune-mediated skin diseases, which affects 3–4% of the human population [1]. Moreover, the etiology of this disease is still not fully understood due to its complex causes, which include both genetic and environmental factors, such as obesity, metabolic/hormonal factors, skin injury, infections, smoking, stress, sunburn and autoimmune reactions. As a result, pathological cytokine-dependent interactions occur between immune cells and epidermal keratinocytes. It is suggested that during psoriasis lesson development, keratinocytes produce a chemotactic gradient by releasing interleukin 8 (IL-8) and growth-regulated oncogene-α (GRO-α), which is indispensable for the migration of neutrophils into the epidermis [2]. Moreover, circulating dendritic cells (DCs) lodge in the epidermis and are strongly activated in psoriatic lesions producing interferon α (IFN-α), tumor necrosis factor α (TNF-α) and interleukins (IL-20 and IL-23), together with enzyme inducible nitric oxide synthase (iNOS) and lymphoid-organizing chemokines such as CCL19, CCL21, CXCL12, and CCL18, that promote T cell activation [[3], [4], [5]]. As a result, secretion of IL-17 increases, which stimulates keratinocytes to produce IL-6, IL-8, platelet-derived growth factor (PDGF), vascular endothelial growth factor (VEGF) and intercellular adhesion molecule-1 [6,7]. Keratinocyte-derived cytokines influence the growth of supporting stromal cells, which in turn overexpress factors such as keratinocyte growth factor (KGF), that induce keratinocyte proliferation [8]. Moreover, many of the above-mentioned cytokines, including IL-6, IL-17, IL-20, IL-22, TNF-α and IFN-α, also stimulate keratinocyte proliferation [2], which in turn activate immune cells [9]. As a result, the normal physiological keratinocyte cycle of differentiation and exfoliation from the epidermis is accelerated from approximately 21 to 4 days [10]. This is caused not only by pro-inflammatory cytokines and chemo-attractants but also by the over-activation of mitogen-activated protein kinases (MAPKs) and up-regulation of redox-sensitive transcription factors (AP-1 and Nrf2) that are involved in the progress of psoriasis at the stage of stimulating hyper-proliferation of skin cells [2]. Although hyper-proliferation mainly refers to epidermal keratinocytes, disturbances in the activity of MAPKs and transcription factors also affect other skin cells, such as fibroblasts, together with cells of the immune system, including neutrophils, lymphocytes and DCs [11]. The chronic inflammation connected with oxidative stress observed in these cells leads to the development of various forms of psoriasis, including the most common psoriasis vulgaris, psoriatic arthritis, or generalized pustular psoriasis [12] (Fig. 1). Moreover, psoriasis can be accompanied by comorbidities, such as insulin resistance, metabolic syndrome, atherosclerosis, arterial hypertension, and depression [13]. This highlights the importance of assessing metabolic/proteomic changes in patients with psoriasis to understand its pathophysiology, but also correlating these results with clinical data for appropriate and early diagnosis and effective therapy.

## 2 Oxidative stress in psoriasis development

Regardless of the above, the described pathological conditions are associated with disordered redox homeostasis and oxidative stress [14,15]. It has been found that psoriasis development is associated with a reduction in the total antioxidant status (TAS) of plasma connected with cellular deficiencies in antioxidant enzyme activity, especially in the case of catalase (CAT) [16]. In addition, these changes, together with the circulating levels of oxidative stress markers (including lipid peroxidation products), make up the psoriasis area severity index (PASI) [16,17]. However, oxidative stress triggered by external factors independently increases the severity of psoriasis [18]. Furthermore, the activities of other antioxidant enzymes, including glutathione peroxidase 4 (GSH-Px4), glutathione reductase (GSSG-R) and thioredoxin reductase (Trx-R), are inhibited in the plasma of patients with psoriasis; the degree of which depends on the type of disease (psoriasis vulgaris vs. psoriatic arthritis) [19,20]. Interestingly, previously published data on superoxidase dismutase (SOD) activity in psoriatic patients has been inconsistent because both its increase [19] and its decrease [21] have been described simultaneously.

Parallel to these changes, also enhanced activity of pro-oxidative enzymes (such as xanthine oxidase), resulting in increased ROS generation has been observed in psoriatic keratinocytes [22]. That causes oxidation of transmembrane proteins, which leads to increased expression of additional pro-inflammatory mediators (IL-1β and IL-6) expression [23], together with oxidative damage. For example, damaged mitochondrial membranes lead to the release of cytochrome *c*, inducing the apoptosis pathways of undifferentiated keratinocytes, the key mechanism of psoriasis [24]. Moreover, other biological compounds, including proteins and lipids, may be oxidized [14]. Lipids may be directly oxidized by ROS, causing the formation of lipid peroxidation products. This can lead to enhanced metabolism with the participation of lipolytic enzymes (PLA2, COXs, LOXs) that are activated under oxidative stress, generating lipid mediators [25], a phenomenon that has been observed in immune cells from psoriatic patients [26]. Examples of such products include different groups of eicosanoids, that are a significant stimulator of keratinocyte proliferation in psoriasis [27,28]. Moreover, oxidative stress leads to the repression of the proteasomal system (mainly based on 20S proteasome) and autophagy, responsible for the degradation of oxidatively modified proteins in immune system cells of patients with psoriasis, that favors NFκB activation and production of pro-inflammatory cytokines [29].

## 3 Lipid peroxidation

During lipid peroxidation, the lipid molecules are oxidized with the formation of lipid radicals. Enzymatic lipid peroxidation is primarily catalyzed by lipoxygenases and cyclooxygenases [30], while non-enzymatic lipid peroxidation is mainly initiated by the hydroxyl and hydroperoxyl radicals. The most common targets for these reactions are phospholipids containing polyunsaturated fatty acids (PUFAs). As a result of the direct ROS reaction with PUFA, a hydrogen atom is detached from the carbon atom from the methylene group adjacent to the double bond with the introduction of oxygen, which causes the formation of a lipid peroxide radical and lipid hydroperoxide generation [31]. The subsequent lipid hydroperoxides undergo further reactions with oxidative fragmentation and the formation of unsaturated α,β-aldehydes, including 4-hydroxynonenal (4-HNE), malondialdehyde (MDA), and acrolein, among others [32]. Moreover, the propagation of the PUFA oxidation chain reaction often leads to oxidative cyclization with the generation of prostaglandin derivatives, including isoprostanes [33], despite of the fact that prostaglandin formation is mainly enzyme-dependent reaction [34]. Lipid peroxidation products directly affect the physical properties and function of the cell membranes within which they are formed. Moreover, regardless of the fact that they are molecules with a relatively short lifetime and high reactivity (connected with their structure: the presence of a carbonyl group and carbon-carbon double bonds), lipid peroxidation products can also act as a signaling molecules [35]. Moreover, by the formation of adducts with the cell's nucleophilic components, including DNA, lipids and proteins, they can significantly affect the functioning of these compounds, leading to metabolic modifications, cellular dysfunction, and even apoptosis [36].

## 4 Adducts of lipid peroxidation products and proteins in psoriasis

### 4.1 4-HNE – protein adducts

One of the best-known and studied products of lipid peroxidation that forms adducts with proteins is 4-HNE [37]. Due to its chemical structure (carbonyl group on the C1 carbon, a double bond between the C2 and C3 carbons, and a hydroxyl group on the C4 carbon), 4-HNE is one of the most reactive electrophilic aldehydes and can form both Schiff bases and Michael adducts with proteins, especially on amino acid residues cysteine, histidine, and lysine [38]. Depending on the protein molecule that is involved in this complex, the resulting 4-HNE-protein adducts have different functions, including pro-oxidant, pro-inflammatory or pro-apoptotic action [37], that can contribute to the development of psoriasis and other skin disorders [39].

In the case of oxidative stress, increased lipid peroxidation (up to 2-times compared to the control) in psoriasis observed as an elevated 4-HNE level has been described in most of the analyzed samples, including whole skin biopsies [40] or various types of cells from the epidermis [22], as well as immune system cells [26,29] and plasma [25]. Additionally, these results in different types of samples are accompanied to the same extent by an increased level of 4-HNE-proteins level [29,41,42]. This leads to significant changes in intercellular signaling that may occur [39] (Table 1) in the psoriatic patient's body.

**Table 1:** Changes in lipid peroxidation products and their adducts with proteins in samples of psoriatic patients. Abbreviations: 4-HNE, 4-hydroxynonenal; 4-ONE, 4-oxononenal; MDA, malondialdehyde.

Sample of psoriasis patients	Lipid peroxidation product	Observation in psoriasis	Modified proteins	Method of detection	Ref.
Skin biopsy	4-HNE	increased level of 4-HNE	–	immune-histochemistry	[40]
	MDA	increased level of MDA (2-times higher vs. control)	–	spectro-photometry	[68]
Keratinocytes	4-HNE	increased level of 4-HNE (2-times higher vs. control)	–	GC-MS	[22]
		increased level of 4-HNE-protein adducts (2-times higher vs. control)	catalase thioredoxin reductase	LC-MS	[41]
	MDA	increased level of MDA (by 20% vs. control)	–	GC-MS	[69,70]
Fibroblasts	MDA	increased level of MDA (by 40% vs. control)	–	GC-MS	[70]
Lymphocytes	4-HNE	increased level of 4-HNE and 4-HNE protein adducts (one-third higher vs. control)	–	GC-MS	[26,29]
				ELISA	
		increased level of 4-HNE-protein adducts (2-times higher vs. control)	transporters, receptors, signal transducers, catalytic enzymes	LC-MS	[41]
	MDA	increased level of MDA (by 30% vs. control)	–	GC-MS	[69]
		increased level of MDA-protein adducts (2-times higher vs. control)	oxidoreductases, hydrolases	LC-MS	[73]
	acrolein	increased level of acrolein-protein adducts	–	western blotting	[82]
	4-ONE	increased level of 4-ONE-protein adducts (by 150% vs. control)	ubiquitin transferase,	LC-MS	[73]
			NFκB repressing factor,		
			14-3-3 protein σ		
Plasma	4-HNE	increased level of 4-HNE and 4-HNE-protein adducts (2-times higher vs. control)	–	GC-MS	[19]
				ELISA	
		increased level of 4-HNE-protein adducts (2-times higher vs. control)	catalytic enzymes	LC-MS	[42]
	MDA	increased level of MDA (2-times higher vs. control)	–	spectro-photometry	[68,71]
		no changes	–	HPLC	[88]

Furthermore, it has been found that 4-HNE can react with receptors, including nuclear hormone receptors, e.g., PPARδ, and induce their activation. However, this receptor activates the transcription of genes that are highly expressed in cancer cells [43], further supporting their effect on the stimulation of cell proliferation. As a result, the expression of keratinocyte differentiation markers such as involucrin and transglutaminase 1 is stimulated, that in the case of psoriasis, intensifies the exfoliation of the epidermis [44].

It is well known that cysteine residues are the most reactive and susceptible to modifications parts of the protein [45]. 4-HNE binding to a cysteine residue of the active site of an antioxidant enzyme, including CAT, glutathione peroxidase and thioredoxin reductase, leads to their inactivation [46,47]. In keratinocytes isolated from psoriatic skin, it has been clearly shown that 4-HNE binds proteins mostly via cysteine, as well as histidine residues, thereby impairing the action of antioxidant enzymes, including CAT and thioredoxin reductase in these cells [41]. Interestingly, this type of change has not been observed for lymphocytes, even though they come into direct contact with skin cells [41]. However, while there is only a small increase in the amount of protein adducts formed in psoriatic lymphocytes compared to controls, the profile of modified proteins is completely different between the two. In control lymphocytes, 4-HNE-protein adducts are created on proteins whose molecular function is based on the binding of other molecules (e.g., immunoglobins), while in psoriatic lymphocytes, such complexes are formed on transporters, receptors, signal transducers, and proteins with catalytic activity [41]. In addition, similar changes have also been observed in the plasma of psoriatic patients, where proteins forming adducts with 4-HNE mainly had catalytic activity, compared to binding molecules that are modified by 4-HNE in controls [42].

Interestingly, 4-HNE can bind to the cysteine residues of the Keap1 protein, which is a cytosolic inhibitor of the antioxidant transcription factor Nrf2 [48]. As a result, Keap1 changes conformation and dissociates Nrf2, then translocates to the nucleus and initiates the biosynthesis of cytoprotective proteins. Moreover, Nrf2 activation pathway may also be associated with the presence of activator proteins such as KAP1, p21 or p62, and inhibitory proteins such as Bach1, which also form adducts with Nrf2 or Keap1. Unfortunately, the exact effect of 4-HNE on their interaction with Nrf2 remains unknown, although 4-HNE promotes Nrf2 activation [49]. Regardless of the good side of the Nrf2 factor, in the case of psoriatic cells, its high activity stimulates cell proliferation and keratinization, causing severe psoriatic skin lesions [26,50]. Despite numerous indications regarding the role of 4-HNE-Keap1 adducts in Nrf2 activation in psoriasis [51], this has not yet been directly determined.

Conversely, and in contrast to other reactive aldehydes, 4-HNE forms adducts that stimulate the pro-inflammatory activity of NFκB [35,52]. Under physiological conditions, NFκB is blocked in the cytoplasm by its natural inhibitor IκB. However, the phosphorylation of IκB causes NFκB dissociation and activation of its transcriptional capacity [53]. 4-HNE can interact with IκB and, following 4-HNE-IκB adduct formation, activate NFκB [54]. This results in the expression of pro-inflammatory cytokines, as well as factor TNFα is increased, which is observed in the case of autoimmune diseases such as psoriasis [55,56].

Moreover, 4-HNE, as a signaling molecule, stimulates cell apoptosis [57], thereby accelerating the exfoliation of the epidermis. 4-HNE and caspase-3, cause the activation of apoptosis [58]. The lack of direct data on the interaction of 4-HNE with caspase 3 in psoriatic patient samples forces us to only reference results stating that enhanced 4-HNE-protein adduct formation, these patients have very high level/activity of this pro-apoptotic protein [59]. Therefore, increased 4-HNE-protein adduct formation can, directly and indirectly, lead to the development of psoriasis.

### 4.2 MDA – protein adducts

Another well-known lipid peroxidation product that can react with proteins is MDA, which shows a high affinity for the formation of adducts with lysine amino acid residues via a Schiff base reaction. As a result, both MDA-lysine adducts or lysine-MDA-lysine cross-links are formed [60]. The formation of MDA-protein adducts is mainly associated with pro-inflammatory reactions [35]. MDA has no effect on biosynthesis/level of protein kinase C (PKC), however by binding to PKC facilitates the activation of this kinase and, as a result, the phosphorylation of the IκB molecule [61]. Phosphorylated IκB no longer plays the role of an NFκB inhibitor, leading to its activation. It has been noted that MDA-protein adducts (among others) lead to the activation of Th17 lymphocytes [62], stimulate the secretion of pro-inflammatory cytokines such as interleukins IL-6, IL-8, and IL-25 [63], and can trigger autoimmune reactions. This can also occur in psoriasis patients, as they are undergoing oxidative stress, lipid peroxidation and even 2-times increased MDA levels [64,65]. However, more data exists on the role of MDA-protein adducts in other skin diseases, such as lupus or melanoma [66,67].

The increase in MDA levels has been documented in a number of different sample types from people with psoriasis (e.g., 2-times in skin biopsy [68], by 20% in keratinocytes [69], by 40% in fibroblasts [70], by 30% in erythrocytes, granulocytes, lymphocytes [69], and 2-times in plasma [68,71]) which are accompanied by further studies indicating the presence of MDA-LDL adducts (low-density lipoprotein) in plasma [72] or increased by 2-times level of MDA-protein adducts in lymphocytes [73] (Table 1). Proteins that structure is most affected by MDA are oxidoreductases and hydrolases. MDA has not been found as a molecule that can change their expression directly, however by affecting the activity of protein kinases, as mentioned above, it is possible that MDA may activate/silence various pathways including specific genes expression. Both types of mentioned enzymes have been found activated in the case of psoriasis [74,75]. Oxidoreductases (e.g., NAD(P)H quinone oxidoreductase 1 (NQO1)) can influence NFκB activation and inflammatory cytokine expression [76], while hydrolases (e.g., acyloxyacyl hydrolase or leukotriene-A4 hydrolase) stimulate psoriatic lymphocytes to inflammatory signaling with interleukin secretion [77,78]. As a result, lymphocytes proceed to induce systemic inflammation, and once they have migrated to the skin, they facilitate keratinocyte hyperproliferation.

### 4.3 Other protein modifications

Other reactive products of lipid peroxidation have been found at increased levels in psoriatic samples [79]. According to their high reactivity, they can also bind to proteins such as the previously described acrolein [35]. Acrolein is an unsaturated aldehyde that can be formed by intracellular metabolism, e.g., during threonine degradation, amine catabolism, and by peroxidation of lipids containing polyunsaturated fatty acids [80]. Cytotoxicity of acrolein is associated with the formation of Michael adducts with thiol groups of cysteines, which may occur spontaneously or may be catalyzed by glutathione S-transferase [81]. As a result, the activity of the modified protein is changed. The level of acrolein-protein adducts is significantly increased in psoriasis patients [82] (Table 1). However, so far, it is not known which specific proteins undergo this modification. Regardless of such data, it is known that protein modifications by acrolein reduce the activity of antioxidant proteins (glutathione, glutathione S-transferase, thioredoxin), leading to increased oxidative stress, and activation of pro-apoptotic pathway kinases, which lead to additional cell death [35]. This is particularly important information in the context of the excessive cell death experienced by keratinocytes in psoriatic skin lessons.

Additionally, in the blood cells of patients with psoriasis, 4-oxononenal (4-ONE)-protein adducts are also increased by 150% comparing to control samples [73]. 4-ONE, similar to 4-HNE, can bind protein cysteine, lysine or histidine residues. In psoriatic lymphocytes, the most strongly modified proteins are the repressive transcription factor NFκB and 14-3-3 protein σ (Table 1). NFκB is known to be involved in the inflammatory response, therefore the modifications of its structure enhance pro-inflammatory signaling, leading to the influx of immune cells into the epidermis and the release of cytokines driving the proliferation of keratinocytes and the intensification of psoriatic lesions. Conversely, 14-3-3 protein σ is a negative regulator of the cell cycle, meaning its impairment will stimulate the cell cycle progression of keratinocytes and their proliferation. Interestingly, 4-ONE has been shown to form adducts with proteins with ubiquitin transferase activity [73]. Ubiquitination in psoriasis plays a primary role due to its effect on NFκB activation and induction of the inflammatory response [83,84]. However, some literature reports show that ubiquitin-modified proteins in psoriatic patients depend on the development of psoriasis vulgaris or psoriatic arthritis [85]. Therefore, having no data on whether the attachment of 4-ONE to ubiquitin transferase causes its activation or inactivation, it is impossible to determine exactly which of these reactions takes place in the case of psoriasis.

Oxidative stress associated with the development of psoriasis leads to numerous oxidative protein modifications, including a whole range of lipid peroxidation products. However, there is a range of other products produced, including pyrolized proteins (PP), protein carbonyl compounds (PCC), methylglyoxal-proteins (MG-protein), advanced oxidation protein products (AOPP), as well as lipid hydroperoxides (LHP) that are increased in the plasma of psoriatic patients [86,87]. As a result, the proteomic profile of psoriatic patients is altered to the degree that it is difficult to trace changes occurring in a single metabolic pathway. Moreover, there are also conflicting reports showing that protein modifications, including lipid peroxidation products, do not occur in psoriasis [88].

### 4.4 Clinical approach - potential therapeutic use of lipid peroxidation products and protein adducts

Protein oxidation, including the formation of protein adducts with lipid peroxidation products (lipoxidation), has been linked to a wide range of pathologies and can be used in the diagnosis and treatment of many diseases [89], especially cardiovascular pathologies [90]. However, lowering the elevated level of reactive aldehyde-protein adducts is, among others, the purpose of pharmacotherapy for diseases associated with aging, such as Alzheimer's and Parkinson's [91]. Covalent modification of mitochondrial biomolecules, including proteins by lipid peroxidation products, has also been recognized as a therapeutic target in the context of cancer pathogenesis [92,93]. Modern molecular therapies focus on disease-specific symptoms, and therefore changes in the formation of lipid peroxidation products and especially their interaction with particular proteins may be bioactive markers that will improve treatment processes [94]. This is also true in the case of psoriasis. This is of particular importance as lipid peroxidation is directly proportional to the PASI [16,17], and therefore the concentration of protein-lipid peroxidation adducts could be a determinant for the choice of appropriate therapy. Conversely, UVB radiation is often used in psoriasis therapy, although it is known to induce lipid peroxidation in skin cells [95] and affect the expression and structure of proteins. This leads to modifications of function, which has consequences for proteins involved in apoptosis, DNA damage response and cell cycle control, angiogenesis, inflammation, mitochondrial biogenesis and keratinocyte differentiation, resulting in effectively calming psoriatic skin lesions [96].

So far, several compounds involved in the inhibition of protein adduct formation are known [97], including DNPH, which prevents formation and accumulation of acrolein and 4-HNE adducts on cellular proteins in vascular cells *in vitro* [98]. However, its application is limited because of its high mutagenic and toxic properties. Vitamin B6 derivatives (pyridoxamine) also can prevent the formation of protein adducts due to the inhibition of lysine modifications [99]. Since lipid peroxidation products are directly caused by the oxidative condition, antioxidants have been studied for the attenuation of reactive aldehyde-protein adducts. In the case of psoriasis, the inhibition of the pro-oxidative enzyme NADPH oxidase has been successfully demonstrated to reduce even by half 4-HNE-protein adduct levels [100]. On the other hand, the activation of the antioxidant/cytoprotective Nrf2 was applied to reduce inflammation in monocytes. However, at the same time, increased psoriatic keratinocyte proliferation has been observed following antioxidant treatment [101].

In the treatment of inflammatory diseases, natural antioxidants can also be used [102]. The natural phytocannabinoid cannabidiol (CBD), due to its antioxidant and anti-inflammatory properties, has been proposed for psoriasis treatment [103]. CBD has been found to be able to significantly protects skin cells against UV-induced oxidative stress, including 4-HNE-proteins adducts formation (prevents their formation by 80%) [104,105]. However, according to the multipolarity of these adducts, CBD significantly reduces the level of 4-HNE but acts selectively on 4-HNE-protein adducts [22]. As a result of CBD application on psoriatic skin, decrease keratinocyte proliferation and a significant reduction of lesions were observed [106,107]. Additionally, CBD also reduces pain in the case of psoriatic arthritis [108]. Other antioxidant and anti-inflammatory compounds are suggested as potential anti-psoriatic factors, whose activities are based on blocking lipid peroxidation products’ interaction with proteins, e.g., lipid extract of microalgae [109], although there is not enough data to confirm this unequivocally.

What deserves additional attention, is the adducts of proteins with products of lipid peroxidation that are also significant when psoriasis occurs in conjunction with comorbidities, including insulin resistance, in which a 40% increase in 4-HNE-protein adducts has been found in plasma [rats model of psoriasis] [110]. Moreover, similar data are observed in atherosclerotic cardiovascular diseases [90,111]. The mechanism of generation and action of these adducts is also suggested for psoriasis-associated depression [112]. The lipid peroxidation product 4-HNE also forms toxic protein adducts with the vesicular monoamine transporter 2 (VMAT2), a membrane protein crucial for the packaging of monoamines into synaptic vesicles. The formation of 4-HNE-VMAT2 adducts prevents the translocation of monoamines [113], while the deficit in monoaminergic neurotransmissions is the most realistic hypothesis regarding the development of depression [114]. Therefore, preventing the formation of lipid peroxidation product-protein adducts formation, as well as modulating their concentrations during psoriasis therapy, can effectively contribute to the treatment of psoriasis-associated diseases.

## 5 Conclusion

Psoriasis is a skin disease that affects the entire human immune system, with all accompanying comorbidities, and is a large burden for patients. Therefore, it is important to study and learn about the complex mechanism of its formation. As we present in this review, changes occurring during the development of psoriasis are facilitated, to a large extent, by lipid peroxidation and its influence on protein structures. It can be quite clearly summarized that these changes concern proteins related to the maintenance of redox homeostasis or pro-inflammatory signaling. Therefore, the formation of such adducts should attract attention, especially during the design of preventive cosmetics or anti-psoriasis therapies.

## Author contributions

Conceptualization: A.W., A.G.; Data curation: A.G.; Methodology: A.W., A.G.; Resources: A.W.; Software: A.G.; Supervision: E.S.; Validation: A.W.; Visualization: A.G.; Writing - original draft: A.W., A.G.; Writing - review & editing: E.S.

## Funding

This review did not receive any specific grant from funding agencies in the public, commercial, or not-for-profit sectors.

## Declaration of competing interest

The authors have no conflicts of interest to declare.

## Data Availability

No data was used for the research described in the article.
